# Increasing Bioactive Compound Production in Lettuce by Application of *Trichoderma* sp. Strain STP8

**DOI:** 10.3390/biom16071073

**Published:** 2026-07-22

**Authors:** Božidar Benko, Mia Dujmović, Sanja Radman, Jana Šic Žlabur, Snježana Topolovec-Pintarić

**Affiliations:** 1 Division of Horticulture and Landscape Architecture, Department of Vegetable Crops, Faculty of Agriculture, University of Zagreb, 10000 Zagreb, Croatia; sradman@agr.hr; 2 Division of Agricultural Engineering, Department of Sustainable Technologies and Renewable Energy Sources, Faculty of Agriculture, University of Zagreb, 10000 Zagreb, Croatia; jszlabur@agr.hr; 3 Division of Phytomedicine, Department of Plant Pathology, Faculty of Agriculture, University of Zagreb, 10000 Zagreb, Croatia; tpintaric@agr.hr

**Keywords:** *Lactuca sativa* L., biostimulants, polyphenols, phenolic compounds, flavonoids and non-flavonoids, antioxidant capacity

## Abstract

Improving the nutritional quality of food through advanced and sustainable agricultural practices has become a key objective of modern vegetable crop production. Emphasis is placed on increasing the content of health-promoting bioactive compounds, such as vitamins and polyphenols, particularly flavonoids whose accumulation is strongly affected by various biotic and abiotic stress factors. To mitigate stress-induced limitations and enhance plant performance, biostimulants are increasingly applied. Among them, *Trichoderma* spp. are widely recognized for their ability to promote plant growth and resilience, primarily through enzymatic activity and the production of bioactive metabolites. The aim of this study was to evaluate the potential of the native *Trichoderma* sp. strain STP8 to enhance the production of bioactive compounds through seed and soil applications at planting and 26 days after planting (DAP), applied individually or in combination. A spore suspension (4 × 10^6^ spores mL^−1^) was used. The experiment was arranged in a randomized complete block design with five replicates. At harvest (43 DAP), dry matter, ascorbic acid, chlorophyll, and carotenoid contents were determined. Additionally, flavonoids and non-flavonoids, total phenolics, individual phenolic compounds, and antioxidant capacity were analyzed. Achieved results demonstrate that the effects of the native *Trichoderma* sp. strain STP8 on lettuce secondary metabolism and antioxidant properties are strongly dependent on the developmental stage at which inoculation is performed, providing further insight into the stage-specific interactions between plans and *Trichoderma*. Practically, a single application at planting proved to be the most effective strategy for enhancing the accumulation of bioactive compounds, indicating that optimized application timing may improve the efficacy of *Trichoderma*-based biostimulants, while avoiding unnecessary repeated applications. These findings support the potential use of native *Trichoderma* strains as sustainable tools for improving the nutritional and functional quality of lettuce. Further research integrating physiological, biochemical, and molecular analyses is required to elucidate the mechanisms by which the native *Trichoderma* sp. strain STP8 regulates the biosynthesis of bioactive compounds in lettuce.

## 1. Introduction

Climate change has become one of the major challenges facing modern agriculture, with increasing temperatures, more frequent droughts, irregular precipitation patterns, and a greater incidence of extreme weather events threatening crop productivity and quality worldwide. These environmental changes not only reduce yields but also increase the occurrence of biotic and abiotic stresses, highlighting the need for sustainable strategies that improve crop resilience while reducing dependence on synthetic agrochemicals.

Lettuce (*Lactuca sativa* L.) is one of the most widely cultivated leafy vegetables worldwide, as evidenced by current production statistics for 2024. It is grown on approximately 1.29 million ha, with a total annual production of around 28.2 million tons (including chicory). In Europe, lettuce is cultivated on approximately 130,358 ha, yielding about 3.46 million tons annually [1]. In 2025, in Croatia, lettuce was grown on 351 ha of open-field land and 51 ha of greenhouse area, with a total annual production of 6659 tons [2]. Besides its economic importance, lettuce is an important component of a healthy diet due to its high content of vitamins, minerals, dietary fiber, and bioactive compounds. Among them, phenolic acids, flavonoids, carotenoids, and other antioxidant molecules contribute significantly to the nutritional and functional value of lettuce by reducing oxidative stress and providing health-promoting properties [3,4,5]. The concentration of these compounds is influenced by several factors, including genotype, environmental conditions, cultivation practices, and biostimulant application [5,6].

Among the sustainable approaches proposed to mitigate the negative impacts of climate change, beneficial microorganisms have attracted considerable attention due to their ability to enhance nutrient uptake, stimulate plant growth, improve tolerance to biotic and abiotic stresses, and reduce dependence on synthetic agrochemicals [7,8,9]. Among them, fungi of the genus *Trichoderma* Pers. (Hypocreales, Ascomycota) are recognized as multifunctional biostimulants and biocontrol agents that colonize plant roots, improve nutrient acquisition and root development, and enhance plant resilience to environmental stresses [10,11].

The beneficial effects of *Trichoderma* are mediated through the production of phytohormones, siderophores, volatile organic compounds, and secondary metabolites together with activation of plant defense signaling pathways and induced systemic resistance [11]. These interactions may stimulate secondary metabolism and enhance the accumulation of antioxidant compounds.

Recent studies have demonstrated that *Trichoderma* inoculation can enhance phenolic compound accumulation and antioxidant activity in several vegetable crops [12,13]. These responses are mainly associated with activation of the phenylpropanoid pathway and modulation of salicylic acid, jasmonic acid, and ethylene-dependent signaling, resulting in increased synthesis of phenolic acids, flavonoids, and other antioxidant metabolites [11,14,15].

Lettuce is particularly suitable for investigating these effects because it naturally contains a diverse spectrum of bioactive compounds whose concentration can be readily influenced by cultivation practices. Previous research [3,16,17] highlighted substantial variability in phenolic content, flavonoid concentration, carotenoid levels, and antioxidant activity among lettuce cultivars, production systems, environmental conditions and stress factors.

Numerous previous studies have already investigated the physiological mechanisms by which *Trichoderma* sp. influence lettuce growth, yield, and development, including effects on germination, reproduction, nutrient uptake, photosynthesis, phytohormone regulation, stress tolerance, and plant defense responses [12,18,19,20,21,22,23]. While these studies have substantially improved our understanding of the role of *Trichoderma* in promoting plant growth and productivity, considerably less attention has been paid to its effects on the lettuce nutritional and functional quality, particularly the accumulation of bioactive compounds. Although Stojanović et al. [24] reported positive effects of a *Trichoderma*-based formulation on lettuce quality and antioxidant activity, the response depended on genotype, fertilizer treatment, and growing season [25,26]. Moreover, the efficacy of *Trichoderma*-based formulations may be reduced by propagule loss during storage and application [27]. Additionally, introduced strains may affect native microbial communities [28,29,30]. Therefore, autochthonous *Trichoderma* strains may provide advantages due to their better adaptation to local environmental conditions.

Understanding the interaction between beneficial fungi and plant secondary metabolism may contribute to the development of sustainable cultivation strategies that improve crop quality and nutritional value. Therefore, the aim of this study was to evaluate the effects of a native *Trichoderma* sp. strain applied at different lettuce growth stages on the accumulation of selected bioactive compounds and antioxidant activity, with particular emphasis on nutritional and biochemical quality. The study specifically focused on the nutritional and biochemical quality of lettuce rather than on elucidating the physiological or molecular mechanisms underlying the observed responses.

## 2. Materials and Methods

### 2.1. Field Trial

The one-year field trial was conducted at the Maksimir Experimental Field (45°49′ N, 16°02′ E), Department of Vegetable Crops, Faculty of Agriculture, University of Zagreb, Croatia. Prior to the experiment, a comprehensive soil analysis was performed using standardized methods [31,32], revealing a neutral soil pH (pH(H_2_O) 7.5; pH(KCl) 6.86), low humus content (2.22%), and adequate nitrogen (0.20% N) and potassium (25.5 mg K_2_O 100 g^−1^ soil) levels, while phosphorus content was high (41.1 mg P_2_O_5_ 100 g^−1^ soil).

The average monthly air temperature from April to July, measured at the Maksimir Meteorological Station [33], ranged from 12.2 to 22.2 °C and remained within the optimal range for outdoor lettuce cultivation. The maximum air temperature reached 27.4 °C. Monthly precipitation was lowest in June (57.7 mm), when irrigation was applied occasionally to maintain optimal soil moisture. Precipitation amounted to 91.6 mm in April and 136.1 mm in May, whereas only 5.0 mm of precipitation was recorded during the first seven days of July.

The native *Trichoderma* sp. strain STP8 used in this study was isolated and identified as described in our previous study [34]. Commercial, untreated seeds of Batavia lettuce (*Lactuca sativa* L.) cv. Bataille (Nunhems Netherlands BV, Nunhems, The Netherlands) were sown on 24 April 2023 in a polystyrene tray containing 104 cells, each filled with 32 mL of autoclaved Potground H substrate (Klasmann-Deilmann, Geeste, Germany). One seed was placed in each cell, after which 0.5 mL of a spore suspension of the strain STP8 (4 × 10^6^ spores mL^−1^) was applied using a micropipette (Eppendorf Research, Stevenage, UK). Seeds that did not receive the spore suspension served as controls and as treatments in which inoculation was omitted.

Seedlings with 3–4 true leaves were manually transplanted into the open field on 25 May 2023 at a spacing of 30 cm within and between rows, resulting in a plant density of approximately 11 plants m^−2^. The experiment was arranged in a randomized complete block design with five replicates, each consisting of 20 plants, resulting in a total of eight treatment variants (Table 1). The STP8 spore suspension, at a concentration of 4 × 10^6^ spores·mL^−1^, was applied at 26 days after planting (DAP) by drenching the soil around the root collar using a knapsack sprayer (Santaj plastika, Valpovo, Croatia). The application times were selected to represent key developmental stages of lettuce: seed sowing, planting, and the intensive vegetative growth stage, to assess whether the response to STP8 depended on the developmental stage at inoculation and the frequency of application, based on previously reported *Trichoderma* application strategies [13,35].

No fertilizers or plant protection products were applied during soil preparation or throughout the experimental period. The plants were harvested on 7 July, 43 DAP. Immediately after harvest, representative samples consisting of three lettuce rosettes from each replicate of each treatment were placed in polyethylene bags and transported in a refrigerated cooler to the Laboratory for Quality Analysis of Agricultural Products of Plant Origin, Faculty of Agriculture, University of Zagreb, where chemical analyses of the plant material were performed. Root samples were also collected after harvest from each treatment and transported to the Laboratory of the Department of Plant Pathology, Faculty of Agriculture, University of Zagreb, to evaluate colonization by strain STP8.

### 2.2. Laboratory Analysis

In this study, well-established and previously standardized analytical laboratory methods were employed and are described only briefly, with references to the original methodological sources provided. All chemicals, reagents, and standards were purchased from Sigma-Aldrich, Merck (St. Louis, MO, USA). All chemical analyses were performed in triplicate.

#### 2.2.1. Determination of Root Colonization

The roots were rinsed with tap water followed by sterile distilled water and placed on sterile filter paper in 20 cm diameter plastic Petri dishes, which served as moist chambers. The samples were incubated at room temperature for seven days, and the presence of STP8 was assessed based on fungal outgrowth from the root samples, following standard procedures for the recovery and identification of *Trichoderma* from plant material [36].

#### 2.2.2. Determination of Total Dry Matter Content

Total dry matter (DM) content was determined according to the official method of the Association of Official Analytical Chemists [37]. Briefly, fresh samples were homogenized, and a known mass was weighed into pre-dried and weighed containers. Samples were dried in a laboratory oven (Memmert, Schwabach, Germany) at 105 °C until constant weight was achieved. After cooling in a desiccator, samples were reweighed. Total dry matter content was expressed as a percentage (%) of the initial fresh mass (fm).

#### 2.2.3. Determination of Ascorbic Acid Content

According to AOAC [37], ascorbic acid (AsA) content was determined by titration using 2,6-dichlorophenolindophenol (DCPIP). Samples were homogenized and extracted for several minutes in oxalic acid. The extracts were filtered and titrated with a standardized DCPIP solution until a persistent light pink endpoint was observed. Ascorbic acid content was calculated using Equation (1) and expressed in mg per 100 g of fresh mass (fm).

(1) AsA (mg/100 g) = (V × F/m) × 100

where V is the volume of DCPIP (mL), F is the factor of the DCPIP (mg·mL^−1^) and m is the sample mass in the filtrate used for titration (g).

#### 2.2.4. Determination of Photosynthetic Pigments

Photosynthetic pigments (chlorophyll a, chlorophyll b, total chlorophylls and total carotenoids) were determined spectrophotometrically. Pigments were extracted from fresh plant material using acetone after which the extracts were filtered. Absorbances were measured at the wavelengths 440, 644, and 662 nm using a UV–Vis spectrophotometer (1900i Shimadzu, Kyoto, Japan). Concentrations of pigments were calculated using Holm–Wettstein equations [38,39] and recalculated in mg per g of fm.

(2) Chl_a = 9.784 × A_662nm_ − 0.990 × A_644nm_ (mg·L^−1^) Chl_b = 21.426 × A_644nm_ − 4.65 × A_662nm_ (mg·L^−1^) TCh = 5.134 × A_662nm_ + 20.436 × A_644nm_ (mg·L^−1^) TCa = 4.695 × A_440nm_ − 0.268 × TCh (mg·L^−1^)

#### 2.2.5. Determination of Total Phenolic Compounds

Total phenolic content (TPC) was determined spectrophotometrically using the Folin–Ciocalteu reagent. Sample extracts were prepared using 80% ethanol (*v*/*v*). An aliquot of the extract was mixed with Folin–Ciocalteu reagent and sodium carbonate solution. After incubation in the dark at room temperature, absorbance was measured at 765 nm. Gallic acid was used as a standard, and results were expressed as mg of gallic acid equivalents (GAEs) per g of sample fm (mg GAE·g^−1^ fm).

Total non-flavonoid content (TNFC) was determined using the formaldehyde precipitation method according to Kramling and Singleton [40]. For the determination of TNFC, 10 mL of the prepared extract was mixed with 5 mL of HCl in EtOH (1:4, *v*/*v*), and 5 mL of formaldehyde (p.a.). The reaction mixture was purged with nitrogen gas and incubated in the dark at room temperature for 24 h to allow flavonoid precipitation. After incubation, the samples were filtered through Whatman filter paper to remove the precipitated flavonoids. The filtrate, containing non-flavonoid phenolic compounds, was analyzed using the Folin–Ciocalteu method, as described above. TFC was calculated from the TPC and TNFC values and expressed as milligrams of catechol equivalents per 100 g fresh mass (mg CTH·100 g^−1^ fm).

#### 2.2.6. Determination of Individual Phenolic Compounds

Individual phenolic compounds were analyzed using high-performance liquid chromatography (HPLC) on an LC Nexera device (Shimadzu, Kyoto, Japan). Plant material was extracted in 80% (*v*/*v*) methanol in an ultrasonic bath. Extracts were filtered through a 0.45 µm membrane filter prior to analysis. Separation was performed on a reverse-phase C18 column using a gradient elution system consisting of acidified water and an organic solvent (e.g., acetonitrile). Detection was carried out using a diode-array detector at multiple wavelengths depending on the compounds of interest. Identification was based on retention times and UV spectra compared to authentic standards, and quantification was performed using external calibration curves. Detailed analysis conditions are listed in the paper by Dujmović et al. [41].

#### 2.2.7. Determination of Antioxidant Capacity

The antioxidant capacity of the samples was evaluated using three complementary spectrophotometric assays, ABTS (2,2′-azino-bis(3-ethylbenzothiazoline-6-sulfonic acid)), DPPH (2,2-diphenyl-1-picrylhydrazyl), and FRAP (Ferric Reducing Antioxidant Power), measured on a 1900i Shimadzu spectrophotometer (Shimadzu, Kyoto, Japan). All results were expressed as µmol Trolox equivalents per g of fresh mass (µmol TE·g^−1^) based on calibration curves constructed with standard Trolox solutions.

The ABTS assay was performed following the method of Miller et al. [42]. The ABTS radical cation (ABTS^•^^+^) was generated by reacting 7 mM ABTS stock solution with 2.45 mM potassium persulfate. The mixture was allowed to stand in the dark at room temperature overnight prior to use. Before analysis, the ABTS^•+^ solution was diluted with 96% ethanol to obtain an absorbance of 0.70 ± 0.02 at 734 nm. An aliquot of the sample extract was mixed with the diluted ABTS^•+^ solution, and the decrease in absorbance was recorded at 734 nm against blank, after 2 min of incubation at room temperature.

The DPPH assay was performed according to Brand-Williams et al. [43] with minor modifications, using a freshly prepared ethanolic solution of DPPH radical (0.1 mM). A defined volume of sample extract was added to the DPPH solution and mixed thoroughly. The reaction mixtures were incubated in the dark at room temperature for 20 min. The decrease in absorbance was then measured at 517 nm against a blank.

Determination of FRAP was done by the method of Benzie and Strain [44]. The FRAP reagent was freshly prepared by mixing 300 mM acetate buffer (pH 3.6), 10 mM TPTZ solution prepared in 40 mM HCl, and 20 mM FeCl_3_·6H_2_O solution in a ratio of 10:1:1 (*v*/*v*/*v*). An aliquot of the sample extract was mixed with distilled water and FRAP reagent, and the mixture was incubated at 37 °C for 5 min. The increase in absorbance due to the formation of the Fe^2+^–TPTZ complex was measured against blank, at 593 nm.

### 2.3. Statistical Analysis

Statistical analyses were performed using SAS^®^ software version 9.4 [45]. Differences among treatments were evaluated by analysis of variance (ANOVA), and treatment means were separated using the LSD test at the 5% significance level (*p* ≤ 0.05).

## 3. Results

### 3.1. Root Colonization with Strain STP8

The novel fungal strain *Trichoderma* sp. STP8 is a native member of the microbiota of the humus garden soil at the Maksimir Experimental Field and was originally isolated from lettuce roots infected with *Sclerotinia sclerotiorum* [34]. Therefore, when recovering the *Trichoderma* sp. STP8 from root samples, we considered the possibility that this fungus could have naturally colonized the lettuce roots.

**Figure 1:**
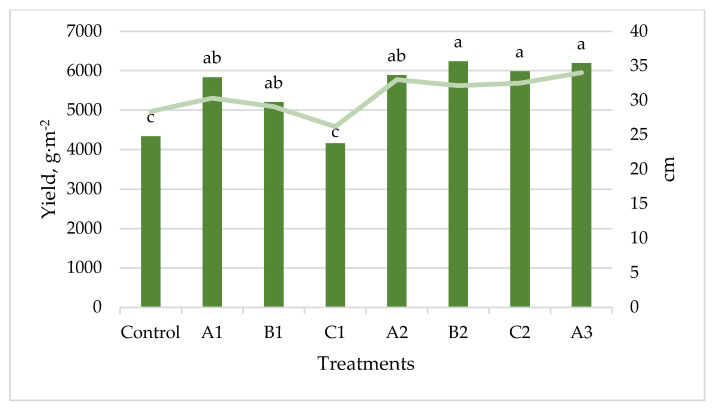
Yield (g·m^−2^) and rosette diameter (cm) of lettuce treated with *Trichoderma* sp. strain STP8. Different letters indicate statistically significant differences between samples at *p* ≤ 0.05. Treatments: Control: untreated plants from untreated seeds; A1: at seed sowing; B1: at planting of seedlings from untreated seeds; C1: 26 DAP seedlings from untreated seeds; A2: at seed sowing and at planting of seedlings; B2: at planting of seedlings (from untreated seeds) and 26 DAP; C2: at seed sowing and 26 DAP; A3: at seed sowing, at planting of seedlings, and 26 DAP.

The tested lettuce cultivar responded well to the inoculation of STP8 in terms of marketable yield. The highest positive effect was achieved with two or three STP8 applications, so the highest marketable yield (6239 g m^−2^) was recorded in treatment B2, followed by treatment A3. All other treatments, except C1 (4160 g m^−2^) and control (4337 g m^−2^), did not differ significantly from B2 and A3. A similar trend was observed for rosette diameter, which ranged from 26.2 cm in C1 to 34.0 cm in A3 (Figure 1).

**Figure 2:**
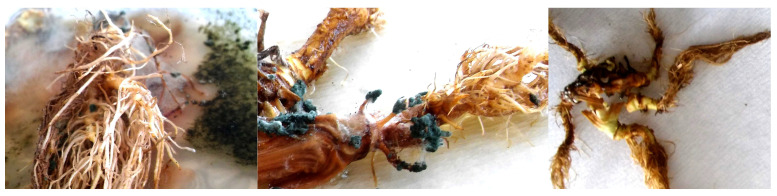
Root samples of lettuce treated with *Trichoderma* sp. strain STP8: variant B2 (**left**); variant A3 (**middle**); control, (**right**).

However, the significant improvements in yield and head diameter observed in the treated plants compared with the untreated control are consistent with successful establishment of strain STP8 and its beneficial interaction with the host plant following spore suspension application (Figure 2).

### 3.2. Total Dry Matter Content

**Figure 3:**
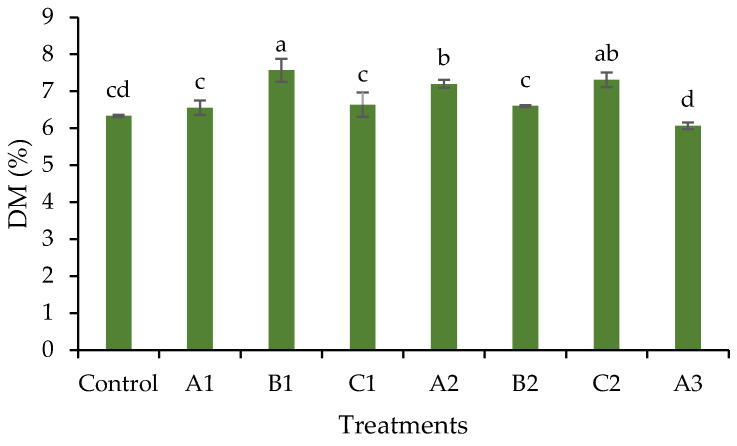
Total dry matter content (DM, %) of lettuce treated with *Trichoderma* sp. strain STP8. Data are represented as mean values ± standard deviation. Different letters indicate statistically significant differences between samples at *p* ≤ 0.05. Treatments: Control: untreated plants from untreated seeds; A1: at seed sowing; B1: at planting of seedlings from untreated seeds; C1: 26 DAP seedlings from untreated seeds; A2: at seed sowing and at planting of seedlings; B2: at planting of seedlings (from untreated seeds) and 26 DAP; C2: at seed sowing and 26 DAP; A3: at seed sowing, at planting of seedlings, and 26 DAP.

The application of the native *Trichoderma* sp. strain STP8 significantly affected the total dry matter (DM) content of lettuce leaves (Figure 3). The highest DM content was recorded in treatment B1 (7.54%), where the spore suspension was applied once at transplanting, followed by treatment C2 (7.28%). Treatment A2 (7.11%) was statistically similar to C2, but like all others significantly lower than B1. In contrast, the lowest DM content was observed in treatment A3 (6.05%), where STP8 was applied three times during the growing period. It was statistically similar to control (6.33%).

### 3.3. Ascorbic Acid Content

**Figure 4:**
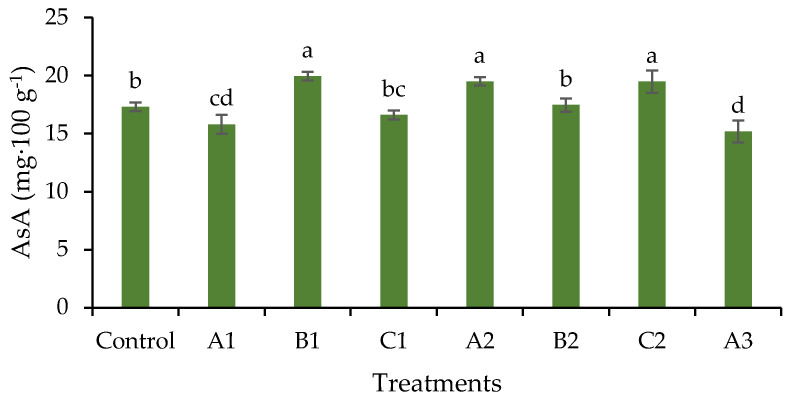
Ascorbic acid content (AsA, mg·100 g^−1^) of lettuce treated with *Trichoderma* sp. strain STP8. Data are represented as mean values ± standard deviation. Different letters indicate statistically significant differences between samples at *p* ≤ 0.05. Treatments: Control: untreated plants from untreated seeds; A1: at seed sowing; B1: at planting of seedlings from untreated seeds; C1: 26 DAP seedlings from untreated seeds; A2: at seed sowing and at planting of seedlings; B2: at planting of seedlings (from untreated seeds) and 26 DAP; C2: at seed sowing and 26 DAP; A3: at seed sowing, at planting of seedlings, and 26 DAP.

The ascorbic acid (AsA) content of lettuce leaves was significantly affected by the application of the native *Trichoderma* sp. strain STP8 (Figure 4). Statistically, the highest AsA concentrations were recorded in treatments B1 (19.94 mg·100 g^−1^), A2 (19.49 mg·100 g^−1^) and C2 (19.48 mg·100 g^−1^). Compared with the control (17.30 mg·100 g^−1^), these treatments increased ascorbic acid content by approximately 13–16%. By contrast, the significantly lowest AsA concentration was observed in treatment A3 (15.17 mg·100 g^−1^) where strain STP8 was applied three times during the growing period. The interesting thing was that it was statistically similar with treatment A1 (15.80 mg·100 g^−1^), i.e., with one application. These findings indicate that increasing the number of applications did not result in a proportional increase in ascorbic acid accumulation and may even have reduced its concentration in lettuce leaves.

### 3.4. Photosynthetic Pigments Content

The results show that the response of photosynthetic pigments to the application of the *Trichoderma* sp. strain STP8 was highly dependent on both the timing and frequency of inoculation (Table 2).

#### 3.4.1. Chlorophyll a

The significantly highest chlorophyll a content was recorded in treatment C1 (0.37 mg·g^−1^), where STP8 was applied once at 26 DAP. It was followed by B2 (0.33 mg·g^−1^) and A1 (0.30 mg·g^−1^), which were significantly lower (Table 2). Compared with the control (0.22 mg·g^−1^), these treatments increased chlorophyll a content by approximately 68%, 50%, and 36%, respectively. By contrast, the lowest values were observed in A3 (0.17 mg·g^−1^) and C2 (0.20 mg·g^−1^), indicating that repeated applications did not promote chlorophyll accumulation.

#### 3.4.2. Chlorophyll b

Chlorophyll b followed a similar trend. The highest content was found in A1 (0.17 mg·g^−1^) and C1 (0.16 mg·g^−1^), which did not differ significantly (Table 2). Treatments B1, A2, and B2 showed intermediate, significantly lower values (0.12–0.13 mg·g^−1^). The lowest content was recorded in the control and C2 treatments (0.09 mg·g^−1^). These results suggest that single applications were generally more effective in stimulating chlorophyll b synthesis than multiple applications.

#### 3.4.3. Total Chlorophyll

Total chlorophyll content ranged from 0.28 to 0.53 mg·g^−1^ (Table 2). The highest value was obtained in C1 (0.53 mg·g^−1^), followed by significantly lower content in the A1 and B2 treatments (0.46 mg·g^−1^). Compared with the control (0.31 mg·g^−1^), these treatments increased total chlorophyll content by approximately 48–71%. Conversely, A3 (0.28 mg·g^−1^) and C2 (0.29 mg·g^−1^) exhibited the lowest total chlorophyll content, even below the control.

#### 3.4.4. Total Carotenoids

The highest carotenoid content was observed in C1 (0.14 mg·g^−1^), followed by a significantly lower content in B2 (0.13 mg·g^−1^) (Table 2). Treatments A1, A2 and the control contained intermediate levels (0.09–0.10 mg·g^−1^), while the lowest carotenoid content was recorded in A3 (0.06 mg·g^−1^). Thus, the increase in chlorophyll content was accompanied by a corresponding increase in carotenoids, suggesting a coordinated enhancement of the photosynthetic apparatus affected by *Trichoderma* sp. strain STP8 application.

**Table 2:** Photosynthetic pigment content (mg·g^−1^) of lettuce treated with *Trichoderma* sp. strain STP8. Data are represented as mean values ± standard deviation. Different superscript letters indicate statistically significant differences between samples at *p* ≤ 0.05. Chl_a—chlorophyll a; Chl_b—chlorophyll b; TChl—total chlorophylls; TCar—total carotenoids.

Treatment	Chl_a	Chl_b	TChl	TCar
Control	0.22 ± 0.01 ^f^	0.09 ± 0.01 ^d^	0.31 ± 0.01 ^e^	0.09 ± 0.01 ^d^
A1	0.30 ± 0.01 ^c^	0.17 ± 0.01 ^a^	0.46 ± 0.01 ^b^	0.10 ± 0.01 ^c^
B1	0.26 ± 0.01 ^d^	0.13 ± 0.01 ^b^	0.39 ± 0.01 ^c^	0.09 ± 0.01 ^d^
C1	0.37 ± 0.01 ^a^	0.16 ± 0.01 ^a^	0.53 ± 0.01 ^a^	0.14 ± 0.01 ^a^
A2	0.24 ± 0.01 ^e^	0.12 ± 0.01 ^b^	0.36 ± 0.01 ^d^	0.09 ± 0.01 ^d^
B2	0.33 ± 0.01 ^b^	0.12 ± 0.01 ^b^	0.46 ± 0.01 ^b^	0.13 ± 0.01 ^b^
C2	0.20 ± 0.01 ^g^	0.09 ± 0.01 ^d^	0.29 ± 0.01 ^f^	0.08 ± 0.01 ^e^
A3	0.17 ± 0.01 ^h^	0.10 ± 0.01 ^c^	0.28 ± 0.01 ^g^	0.06 ± 0.01 ^f^
*p*	≤0.0001	≤0.0001	≤0.0001	≤0.0001
LSD	0.005	0.0071	0.0093	0

Treatments: Control: untreated plants from untreated seeds; A1: at seed sowing; B1: at planting of seedlings from untreated seeds; C1: 26 DAP seedlings from untreated seeds; A2: at seed sowing and at planting of seedlings; B2: at planting of seedlings (from untreated seeds) and 26 DAP; C2: at seed sowing and 26 DAP; A3: at seed sowing, at planting of seedlings, and 26 DAP.

### 3.5. Phenolic Compound Content

The achieved results indicate that the application of *Trichoderma* sp. STP8 significantly affected the accumulation of phenolic compounds in lettuce, although the response strongly depended on the timing and frequency of application (Table 3).

#### 3.5.1. Total Phenolic Compound Content (TPC)

The highest TPC was recorded in B1 (150.40 mg GAE·100 g^−1^), where *Trichoderma* sp. strain STP8 was applied once at transplanting. This treatment increased TPC by approximately 18.5% compared with the control (126.96 mg GAE·100 g^−1^). The second-highest value was observed in A2 (135.02 mg GAE·100 g^−1^), while the lowest TPC was recorded in A1 (67.36 mg GAE·100 g^−1^), representing a reduction of almost 47% relative to the control. Treatments C1, B2, C2, and A3 also exhibited significantly lower TPC values than the control (Table 3).

#### 3.5.2. Total Non-Flavonoid Compound Content (TNFC)

A similar pattern was observed for TNFC. The highest content was found in control (71.31 mg GAE·100 g^−1^) and B1 (70.62 mg GAE·100 g^−1^), which did not differ significantly (Table 3). Treatment A2 (65.77 mg GAE·100 g^−1^) also maintained a relatively high, but significantly lower TNFC level. At the same time, the lowest TNFC was recorded in the A1 (32.27 mg GAE·100 g^−1^), and B2 treatment (41.95 mg GAE·100 g^−1^). These results suggest that seed treatment alone was not effective in stimulating the accumulation of non-flavonoid phenolics.

#### 3.5.3. Total Flavonoid Content (TFC)

Flavonoids showed the most pronounced response to the application of *Trichoderma* sp. strain STP8 (Table 3). Treatment B1 (79.78 mg CTH·100 g^−1^) exhibited the highest TFC, followed by A2 (69.25 mg CTH·100 g^−1^). Compared with the control (55.65 mg CTH·100 g^−1^), these treatments increased flavonoid content by approximately 43% and 24%, respectively. The significantly lowest TFC value was observed in A1 (35.09 mg CTH·100 g^−1^). Also, significantly lower CTH values (12–31% lower) than in control were determined in the C1, B2, C2, and A3 treatments (Table 3).

**Table 3:** Total phenolic compound content (TPC, mg GAE·100 g^−1^), total non-flavonoid compound content (TNFC, mg GAE·100 g^−1^) and total flavonoid content (TFC, mg CTH·100 g^−1^) of lettuce treated by *Trichoderma* sp. strain STP8. Data are represented as mean values ± standard deviation. Different superscript letters indicate statistically significant differences between samples at *p* ≤ 0.05.

Treatment	TPC	TNFC	TFC
Control	126.96 ± 1.49 ^c^	71.31 ± 1.65 ^a^	55.65 ± 2.97 ^c^
A1	67.36 ± 0.34 ^g^	32.27 ± 2.02 ^f^	35.09 ± 2.27 ^e^
B1	150.40 ± 0.98 ^a^	70.62 ± 0.99 ^a^	79.78 ± 0.41 ^a^
C1	88.17 ± 2.4 ^f^	49.71 ± 2.14 ^d^	38.45 ± 1.6 ^e^
A2	135.02 ± 2.81 ^b^	65.77 ± 2.58 ^b^	69.25 ± 4.92 ^b^
B2	86.74 ± 1.54 ^f^	41.95 ± 2.74 ^e^	44.79 ± 4.02 ^d^
C2	108.53 ± 1.52 ^d^	59.47 ± 1.14 ^c^	49.06 ± 2.1 ^d^
A3	99.95 ± 0.7 ^e^	50.90 ± 0.67 ^d^	49.05 ± 0.35 ^d^
*p*	≤0.0001	≤0.0001	≤0.0001
LSD	2.8781	3.2522	4.8026

Treatments: Control: untreated plants from untreated seeds; A1: at seed sowing; B1: at planting of seedlings from untreated seeds; C1: 26 DAP seedlings from untreated seeds; A2: at seed sowing and at planting of seedlings; B2: at planting of seedlings (from untreated seeds) and 26 DAP; C2: at seed sowing and 26 DAP; A3: at seed sowing, at planting of seedlings, and 26 DAP.

### 3.6. Individual Phenolic Compound Content

These results reveal that the effect of the native *Trichoderma* sp. strain STP8 on individual phenolic compounds was highly compound-specific and strongly dependent on the timing and frequency of application. While some phenolics were markedly stimulated, others decreased in response to inoculation, indicating substantial modifications of lettuce secondary metabolism (Table 4).

#### 3.6.1. Phenolic Acids

Among the analyzed phenolic acids, treatment B1 (single application at transplanting) consistently produced the highest concentrations of several important compounds. Chlorogenic acid increased from 8.42 mg·100 g^−1^ in the control to 12.14 mg·100 g^−1^, representing an increase of approximately 44% (Table 4). Similarly, ferulic acid reached its maximum value in B1 (17.98 mg·100 g^−1^), almost doubling the concentration observed in the control (10.33 mg·100 g^−1^). Gallic acid and hydroxybenzoic acid also achieved their highest concentrations in B1, at 2.55 and 1.65 mg·100 g^−1^, respectively. Conversely, other phenolic acids exhibited an opposite trend. The highest contents of protocatechuic acid were recorded in A1 and B2 (10.14 and 10.37 mg·100 g^−1^), whereas B1 exhibited significantly lower level (8.36 mg·100 g^−1^) than the control (10.05 mg·100 g^−1^). Similarly, vanillic acid was highest in B2 and the control (0.94 and 0.83 mg·100 g^−1^) but decreased markedly in B1 (0.59 mg·100 g^−1^) (Table 4).

#### 3.6.2. Flavonoids

The most remarkable response was observed for naringin and luteolin-7-glucoside. Treatment B1 resulted in a significant increase in both compounds, reaching 32.15 mg·100 g^−1^ and 33.70 mg·100 g^−1^, respectively. Compared with the control, naringin increased by approximately 250%, while luteolin-7-glucoside increased by nearly 290%. Such pronounced increases strongly indicate that inoculation at transplanting stimulated flavonoid biosynthesis.

Unlike most other flavonoids, quercetin exhibited a different pattern (Table 4). The significantly highest content was recorded in C1 (13.09 mg·100 g^−1^), followed by A3 and the control, whereas B1 and B2 showed the significantly lowest values (3.39 mg·100 g^−1^). Rutin trihydrate was much less responsive to the treatments with *Trichoderma* sp. strain STP8. Values ranged between 7.04 and 7.26 mg·100 g^−1^, indicating that rutin biosynthesis was relatively stable regardless of inoculation strategy. Nevertheless, B1 again produced the significantly highest content (Table 4).

**Table 4:** Individual phenolic compound content (mg·100 g^−1^) of lettuce treated by *Trichoderma* sp. strain STP8. Data are represented as mean values ± standard deviation. Different superscript letters indicate statistically significant differences between samples at *p* ≤ 0.05.

Treatment	Chlorogenic Acid	Ferulic Acid	Gallic Acid	Hydroxybenzoic Acid	Protocatechuic Acid	Vanillic Acid	Naringin	Luteolin 7 Glucoside	Quercetin	Rutin Trihydrate
Control	8.42 ± 0.25 ^e^	10.33 ± 0.14 ^d^	2.20 ± 0.14 ^d^	0.49 ± 0.17 ^e^	10.05 ± 0.01 ^b^	0.83 ± 0.05 ^a^	9.20 ± 0.19 ^d^	8.73 ± 0.01 ^b^	10.71 ± 0.07 ^c^	7.06 ± 0.01 ^cd^
A1	9.47 ± 0.01 ^d^	10.14 ± 0.03 ^d^	2.31 ± 0.06 ^c^	0.85 ± 0.01 ^c^	10.41 ± 0.09 ^a^	0.81 ± 0.01 ^ab^	10.01 ± 0.02 ^cd^	6.79 ± 0.01 ^d^	10.62 ± 0.01 ^d^	7.06 ± 0.01 ^d^
B1	12.14 ± 0.07 ^a^	17.98 ± 1.3 ^a^	2.55 ± 0.01 ^a^	1.65 ± 0.01 ^a^	8.36 ± 0.09 ^e^	0.59 ± 0.02 ^cd^	32.15 ± 1.72 ^a^	33.70 ± 0.27 ^a^	3.39 ± 0.01 ^g^	7.26 ± 0.02 ^a^
C1	10.40 ± 0.02 ^b^	14.64 ± 0.16 ^b^	2.45 ± 0.01 ^b^	1.05 ± 0.01 ^b^	9.76 ± 0.02 ^c^	0.81 ± 0.01 ^ab^	13.20 ± 0.07 ^b^	6.25 ± 0.01 ^e^	13.09 ± 0.09 ^a^	7.07 ± 0.01 ^cd^
A2	6.80 ± 0.01 ^f^	8.41 ± 0.07 ^e^	2.27 ± 0.01 ^cd^	0.40 ± 0.01 ^e^	10.12 ± 0.22 ^b^	0.42 ± 0.18 ^d^	9.37 ± 0.01 ^cd^	1.04 ± 0.01 ^g^	9.62 ± 0.04 ^e^	7.04 ± 0.01 ^f^
B2	10.18 ± 0.11 ^c^	10.32 ± 0.07 ^d^	2.30 ± 0.01 ^c^	0.03 ± 0.01 ^f^	10.37 ± 0.12 ^a^	0.94 ± 0.07 ^a^	9.97 ± 0.01 ^cd^	7.34 ± 0.02 ^c^	3.39 ± 0.01 ^g^	7.05 ± 0.01 ^e^
C2	8.39 ± 0.01 ^e^	8.07 ± 0.01 ^e^	2.19 ± 0.01 ^d^	0.70 ± 0.05 ^d^	6.61 ± 0.011 ^f^	0.48 ± 0.02 ^cd^	10.39 ± 0.01 ^c^	6.33 ± 0.01 ^e^	9.48 ± 0.01 ^f^	7.14 ± 0.01 ^b^
A3	8.37 ± 0.02 ^e^	11.69 ± 0.05 ^c^	2.33 ± 0.01 ^c^	0.07 ± 0.01 ^f^	9.47 ± 0.02 ^d^	0.64 ± 0.21 ^bc^	10.33 ± 0.04 ^c^	3.87 ± 0.02 ^f^	11.44 ± 0.03 ^b^	7.07 ± 0.01 ^c^
*p*	≤0.0001	≤0.0001	≤0.0001	≤0.0001	≤0.0001	≤0.0001	≤0.0001	≤0.0001	≤0.0001	≤0.0001
LSD	0.176	0.8085	0.0959	0.1111	0.1736	0.1779	1.0598	0.1671	0.0776	0.0079

Treatments: Control: untreated plants from untreated seeds; A1: at seed sowing; B1: at planting of seedlings from untreated seeds; C1: 26 DAP seedlings from untreated seeds; A2: at seed sowing and at planting of seedlings; B2: at planting of seedlings (from untreated seeds) and 26 DAP; C2: at seed sowing and 26 DAP; A3: at seed sowing, at planting of seedlings, and 26 DAP.

### 3.7. Antioxidant Capacity

The results presented in Table 5 demonstrate that the antioxidant activity of lettuce was significantly influenced by the application of the native *Trichoderma* sp. strain STP8, although the response varied among the antioxidant assays used. The differences observed among ABTS, DPPH, and FRAP values indicate that applied strain affected not only the concentration but also the composition of antioxidant compounds present in lettuce leaves.

ABTS values ranged from 22.48 to 25.17 µmol TE·g^−1^. The highest antioxidant activity was recorded in B1 (25.17), although it did not differ significantly from the control, A2, B2, and C2 treatments. The lowest ABTS value was observed in A1 (22.48), indicating that seed treatment alone significantly reduced antioxidant capacity (Table 5). Overall, ABTS activity showed relatively small variations among treatments, suggesting that the total pool of radical-scavenging compounds remained relatively stable despite changes in individual phenolic compounds.

DPPH activity exhibited much greater variation among treatments. The highest value was observed in B1 (6.32 µmol TE·g^−1^), which was almost twice the value recorded in the control (3.31 µmol TE·g^−1^) and nearly four times higher than in A1 (1.67 µmol TE·g^−1^). Treatment A2 also increased DPPH activity, whereas A1, C1, and B2 significantly reduced radical-scavenging capacity, compared to control (Table 5). The performance of the B1 treatment indicates that inoculation at transplanting strongly enhanced the accumulation of compounds with high hydrogen-donating and free-radical-scavenging activity.

The FRAP assay revealed the clearest treatment effects. The highest reducing power was again observed in B1 (16.60 µmol TE·g^−1^), significantly exceeding the control treatment (14.41 µmol TE·g^−1^). Conversely, A1 treatment exhibited the lowest value among all treatments (6.08 µmol TE·g^−1^), corresponding to a reduction of almost 58% compared with the control. Treatments C1, B2, C2, and A3 also showed significantly lower FRAP values than control. These findings suggest that STP8 application at transplanting enhanced the accumulation of compounds capable of electron donation and metal ion reduction.

**Table 5:** Antioxidant capacity (µmol TE·g^−1^) of lettuce treated with *Trichoderma* sp. strain STP8. Data are represented as mean values ± standard deviation. Different superscript letters indicate statistically significant differences between samples at *p* ≤ 0.05.

Treatment	ABTS	DPPH	FRAP
Control	24.86 ± 0.08 ^a^	3.31 ± 0.06 ^c^	14.41 ± 0.05 ^b^
A1	22.48 ± 0.87 ^c^	1.67 ± 0.20 ^f^	6.08 ± 0.07 ^h^
B1	25.17 ± 0.12 ^a^	6.32 ± 0.02 ^a^	16.60 ± 0.07 ^a^
C1	23.78 ± 1.09 ^b^	2.56 ± 0.08 ^e^	8.84 ± 0.07 ^f^
A2	24.92 ± 0.03 ^a^	3.92 ± 0.31 ^b^	13.69 ± 0.09 ^c^
B2	24.70 ± 0.28 ^a^	2.47 ± 0.11 ^e^	8.25 ± 0.08 ^g^
C2	24.86 ± 0.03 ^a^	3.25 ± 0.16 ^c^	11.31 ± 0.07 ^d^
A3	24.57 ± 0.36 ^ab^	2.89 ± 0.02 ^d^	10.14 ± 0.10 ^e^
*p*	0.0002	≤0.0001	≤0.0001
LSD	0.90	0.263	0.13

Treatments: Control: untreated plants from untreated seeds; A1: at seed sowing; B1: at planting of seedlings from untreated seeds; C1: 26 DAP seedlings from untreated seeds; A2: at seed sowing and at planting of seedlings; B2: at planting of seedlings (from untreated seeds) and 26 DAP; C2: at seed sowing and 26 DAP; A3: at seed sowing, at planting of seedlings, and 26 DAP.

## 4. Discussion

### 4.1. Total Dry Matter

Increasing the number of *Trichoderma* sp. strain STP8 applications did not result in a proportional increase in dry matter accumulation. While application at transplanting (B1) and double application at sowing and transplanting (A2) or at seed sowing and 26 DAP (C2) increased DM content compared with the control, three consecutive applications (A3) reduced DM accumulation. This suggests that the timing of inoculation may be more important than the total number of applications.

The increase in dry matter observed in B1, A2 and C2 may be associated with enhanced nutrient uptake and improved physiological activity induced by *Trichoderma*, as previously reported [11,46]. Root colonization by *Trichoderma* can stimulate root growth and nutrient acquisition, resulting in greater synthesis and accumulation of structural and storage compounds in plant tissues. Similar positive effects of *Trichoderma* on lettuce growth and quality were reported by Alzyoud et al. [13], who observed improved growth performance following inoculation with *T. harzianum*.

However, the reduction in dry matter content observed in treatment A3 may indicate that repeated applications stimulated vegetative growth and water accumulation to a greater extent than dry biomass formation. Similar observations have been reported in studies showing that the effects of *Trichoderma* depend on strain characteristics, application strategy, environmental conditions, and plant genotype [24,46,47]. Therefore, the results suggest that moderate application frequencies may be more effective for increasing dry matter accumulation than repeated inoculations throughout the growing period.

### 4.2. Ascorbic Acid Content

Interestingly, single applications at sowing (A1) or at 26 days after planting (C1) resulted in lower AsA contents than the control, whereas application at transplanting (B1) produced the highest value. Since transplanting represents a critical stage characterized by physiological stress and root establishment, inoculation with *Trichoderma* sp. strain STP8 at this stage may have enhanced root colonization, nutrient acquisition, and activation of antioxidant metabolism, which consequently improves lettuce nutritional quality.

Increased AsA accumulation following *Trichoderma* inoculation has been reported in several horticultural crops and is frequently associated with enhanced plant physiological status and reduced oxidative stress [48,49,50]. For example, Rouphael et al. [49] observed that *Trichoderma virens* increased AsA content in lettuce by 61–91% compared with untreated plants. Likewise, inoculation with *Trichoderma harzianum* enhanced AsA accumulation in broccoli [51], while increased AsA concentrations were also reported in *Brassica rapa* treated with *T. harzianum* TM10 [52]. Fungi of the genus *Trichoderma* are known to stimulate plant antioxidant systems through improved nutrient uptake, enhanced photosynthetic activity, and modulation of plant defense responses [11,46]. Therefore, these responses may be associated with *Trichoderma*-induced improvements in nutrient uptake and modulation of the antioxidant system, which can promote both the biosynthesis and regeneration of ascorbate under normal and stress conditions [10,53,54]. In addition, *Trichoderma* strains are known to alter plant gene expression, thereby influencing AsA levels by modulating the enzymes responsible for its biosynthesis [55]. The superior performance of treatments B1, A2 and C2 suggests that one or two applications at key developmental stages are the most effective. Overall, the timing of the application of *Trichoderma* sp. strain STP8 was a key factor influencing ascorbic acid accumulation in lettuce, with inoculation at transplanting producing the most pronounced positive effect.

### 4.3. Photosynthetic Pigments

The results demonstrate that the application of the native *Trichoderma* sp. strain STP8 significantly affected the accumulation of photosynthetic pigments in lettuce. The most pronounced positive effect was observed in treatment C1, where STP8 was applied once at 26 days after planting, resulting in the highest contents of chlorophyll a, total chlorophyll, and carotenoids. Treatments A1 and B2 also enhanced pigment accumulation compared with the control. In contrast, treatments involving repeated applications, particularly A3 and C2, resulted in reduced pigment concentrations, indicating that excessive inoculation did not further stimulate photosynthetic pigment synthesis.

Enhanced chlorophyll accumulation following *Trichoderma* inoculation has frequently been associated with improved nutrient acquisition, especially nitrogen and magnesium uptake, which are essential components of chlorophyll molecules. In addition, *Trichoderma* spp. are known to improve root development and plant physiological status, and enhance photosynthetic efficiency thereby promoting pigment biosynthesis [48,56]. More specifically, *Trichoderma* has been reported to modulate phytohormone signaling, promote chloroplast development, and upregulate the expression of photosynthesis-related genes, including those encoding key enzymes of the Calvin cycle [56,57,58]. Increased chlorophyll and carotenoid concentrations have been reported in several crops treated with *Trichoderma*-based biostimulants and are generally considered indicators of enhanced photosynthetic capacity and plant vigor [56,59].

The superior performance of the C1 treatment suggests that application during the active vegetative growth phase may represent the most effective timing for stimulating pigment biosynthesis in lettuce. Conversely, the reduced pigment content observed in A3 indicates that repeated applications may alter plant metabolism in a manner that does not favor further chlorophyll accumulation. These findings are in accordance with the results of Vukelić et al. [60] and confirm that *Trichoderma* sp. strain STP8 acts more effectively as a targeted biostimulant than as a repeatedly applied inoculant.

### 4.4. Phenolic Compounds

The application of the native *Trichoderma* sp. strain STP8 significantly affected phenolic compound accumulation in lettuce. The most favorable response was observed in treatment B1, where a single application at transplanting resulted in the highest concentrations of total phenolic content (TPC), total flavonoid content (TFC), and non-flavonoid phenolic content (TNFC). Treatment A2 also promoted phenolic compounds accumulation, although to a lesser extent.

While the B1 treatment produced the highest phenolic concentrations, seed treatment alone (A1) resulted in the lowest values for TPC, TNFC, and TFC. Continuous application through the growing period (A3) did not further enhance phenolic accumulation. These findings suggest that transplanting represents a critical stage for effective root colonization and activation of phenolic biosynthesis, i.e., the optimal stage for STP8 application, and that seed inoculation alone is insufficient to stimulate phenolic metabolism in lettuce.

The native *Trichoderma* sp. strain STP8 also significantly altered the individual phenolic profile of lettuce, with the strongest response observed in treatment B1, where a single application at transplanting increased the concentrations of chlorogenic acid, ferulic acid, gallic acid, hydroxybenzoic acid, naringin, luteolin-7-glucoside, and rutin. Chlorogenic acid and its derivatives are among the predominant phenolic compounds in lettuce and contribute substantially to its antioxidant capacity [13,61]. Therefore, the increased accumulation of chlorogenic and ferulic acids observed in B1 may partly explain the enhanced antioxidant activity recorded in this treatment.

The marked increase in naringin and luteolin-7-glucoside suggests that *Trichoderma* sp. strain STP8 influenced flavonoid biosynthesis. Similar changes in flavonoid composition have been reported in lettuce exposed to biotic elicitors and beneficial microorganisms, which can stimulate the production of secondary metabolites involved in plant defense and stress adaptation [62,63]. Since flavonoids are among the most important antioxidants in leafy vegetables, their increased accumulation contributes to improved nutritional quality.

However, the response was highly compound-specific. While chlorogenic acid, ferulic acid, naringin, and luteolin-7-glucoside increased, quercetin, protocatechuic acid, and vanillic acid decreased or remained unchanged. Similar changes in the relative abundance of individual phenolics have been reported in metabolomic studies of lettuce, indicating that environmental and biological factors may redirect carbon flux toward specific branches of the phenylpropanoid pathway rather than uniformly increasing all phenolic compounds [61,64], which is in accordance with our results.

Despite increasing interest in the use of *Trichoderma* spp. as plant biostimulants, knowledge regarding their effects on the quantitative and qualitative composition of phenolic compounds remains limited. In the present study, the observed increase in TPC, TFC and some individual phenolic compounds may be associated with the ability of *Trichoderma* spp. to stimulate secondary metabolism through activation of the phenylpropanoid pathway, thereby enhancing the biosynthesis of phenolic acids and flavonoids [11,46]. In addition, *Trichoderma*-mediated activation of plant defense responses may promote antioxidant metabolism and stimulate key enzymes involved in phenylpropanoid biosynthesis. By secreting bioactive metabolites, *Trichoderma* triggers systemic defense responses that activate phenylalanine ammonia-lyase (PAL), a key enzyme in the phenylpropanoid pathway, resulting in the accumulation of antimicrobial flavonoids and phenolic acids that contribute to both disease resistance and improved nutraceutical quality [65,66,67]. Furthermore, modulation of phytohormone signaling pathways, particularly those involving salicylic acid, jasmonic acid, and ethylene, has also been proposed to regulate secondary metabolite production in *Trichoderma*-treated plants [11,15]. Previous studies have likewise demonstrated that *Trichoderma* significantly influences the biosynthesis of phenolic compounds in plants and may even induce the *de novo* synthesis of phenolic compounds [21,65,66,67]. Similar responses have been reported in several horticultural crops and are generally associated with enhanced antioxidant capacity and improved nutritional quality [18,68]. In lettuce, Rouphael et al. [49] reported a 14% increase in total phenolic content following inoculation with *Trichoderma virens*, while Metwally et al. [21] observed enhanced accumulation of total phenolics and flavonoids in lettuce treated with *T. asperellum*, consistent with the findings of the present study. Likewise, Marra et al. [67] demonstrated increased accumulation of flavonoids, including luteolin derivatives, in olive leaves following *Trichoderma* application, whereas Nawrocka et al. [66] reported significant increases in the concentrations of 23 individual phenolic compounds in cucumber treated with *Trichoderma atroviride* TRS25. Together, these findings support the hypothesis that *Trichoderma* spp. promote phenolic compound accumulation across different plant species, although the magnitude and composition of the response depend on the fungal strain, plant species, and experimental conditions.

### 4.5. Antioxidant Capacity

The antioxidant assays consistently identified treatment B1 as the most effective application strategy for enhancing antioxidant activity in lettuce. A single application of Trichoderma sp. strain STP8 at transplanting resulted in the highest ABTS, DPPH, and FRAP values, corresponding with the increased concentrations of total phenolics, total flavonoids, chlorogenic acid, ferulic acid, naringin, and luteolin-7-glucoside observed in the same treatment. Since phenolic compounds are among the major contributors to the antioxidant potential of lettuce, these results suggest that inoculation with STP8 stimulated secondary metabolism, most likely through activation of the phenylpropanoid pathway, thereby increasing the synthesis of antioxidant metabolites. Similar physiological responses have been reported following *Trichoderma* colonization of plant roots, where fungal metabolites and signaling molecules induce systemic resistance, enhance nutrient uptake, improve photosynthetic efficiency, and stimulate antioxidant metabolism through modulation of signaling pathways with enhanced accumulation of metabolites involved in the phenylpropanoid pathway such as cinnamic acid, coumaric acid, salicylic acid and other phenolic acids and flavonoids [11,15,46].

The higher values recorded in treatment B1 suggest that *Trichoderma* sp. strain STP8 promoted the accumulation of antioxidant metabolites and strengthened the antioxidant defense system of lettuce. Previous studies have shown that Trichoderma spp. can improve plant physiological status by enhancing ROS scavenging capacity and reducing oxidative damage, leading to increased antioxidant activity and stress tolerance [69,70]. These mechanisms likely contributed to the enhanced antioxidant capacity observed in treatment B1, where improved physiological status was accompanied by increased accumulation of phenolic antioxidants.

Although all three assays evaluate antioxidant capacity, they are based on different reaction mechanisms and therefore respond differently to changes in antioxidant composition [71]. The ABTS assay measures the ability of antioxidants to quench the ABTS•+ radical through both electron-transfer and hydrogen atom-transfer mechanisms and can detect both hydrophilic and lipophilic antioxidants. Consequently, ABTS values showed only modest differences among treatments, suggesting that the total pool of radical-scavenging compounds remained relatively stable despite substantial changes in the concentrations of individual phenolics. By contrast, the DPPH assay primarily reflects the hydrogen-donating capacity of antioxidants and is particularly sensitive to specific phenolic compounds with strong radical-scavenging activity. The pronounced increase in DPPH activity observed in treatment B1 therefore corresponds well with the substantial accumulation of chlorogenic acid, ferulic acid, naringin, and luteolin-7-glucoside, all of which possess high hydrogen atom-donating capacity. The FRAP assay is based on a different principle, measuring the reducing power of antioxidants through electron transfer to the Fe^3+^-TPTZ complex. Therefore, FRAP preferentially reflects the concentration of compounds with strong reducing potential rather than radical-scavenging activity alone. The significantly higher FRAP values in B1 indicate an increased abundance of electron-donating antioxidants, which is consistent with the elevated concentrations of hydroxycinnamic acids and flavonoids observed in this treatment.

Conversely, treatment A1, which involved seed application alone, consistently exhibited the lowest antioxidant activity in all three assays. This finding closely corresponds with the reduced concentrations of total phenolics and flavonoids observed in the same treatment, suggesting that seed inoculation alone was insufficient to establish sustained root colonization or induce long-term metabolic responses. In contrast, inoculation at transplanting appears to coincide with a developmental stage during which plant–fungal interactions most effectively stimulate secondary metabolism and antioxidant biosynthesis.

## 5. Conclusions

The present one-year study demonstrated that the novel native *Trichoderma* sp. strain STP8 significantly affected the accumulation of bioactive compounds and antioxidant activity in lettuce. However, the intensity of the response strongly depended on the timing and frequency of application.

Among the treatments applied, a single application at transplanting (B1) proved to be the most effective. This treatment significantly increased the contents of total phenolics, flavonoids, chlorogenic acid, ferulic acid, gallic acid, hydroxybenzoic acid, naringin, luteolin-7-glucoside, and ascorbic acid. Furthermore, treatment B1 exhibited the highest antioxidant activity as determined by DPPH and FRAP assays, indicating an improvement in the nutritional and functional quality of lettuce. Enhanced accumulation of chlorophylls and carotenoids following selected *Trichoderma* sp. strain STP8 treatment also suggests a positive effect on plant physiological status and photosynthetic capacity.

The results may indicate that inoculation stimulated the secondary metabolism, particularly the phenylpropanoid pathway, leading to increased biosynthesis of antioxidant compounds. However, the response was compound-specific, as certain phenolics, including quercetin, protocatechuic acid, and vanillic acid, were either reduced or unaffected by inoculation. These findings suggest that *Trichoderma* sp. strain STP8 modified the allocation of metabolic flux within phenolic biosynthetic pathways rather than uniformly increasing all metabolites.

In contrast, seed treatment alone (A1) generally resulted in the lowest values of phenolic compounds and antioxidant activity, while repeated applications throughout the growing period (A3) did not provide additional benefits compared with a single application at transplanting. Therefore, the effectiveness of STP8 was determined primarily by the developmental stage at which inoculation was performed rather than by the number of applications.

The results demonstrate that the native *Trichoderma* sp. strain STP8 has considerable potential as a biostimulant for improving the nutritional quality and antioxidant properties of lettuce. Application at transplanting proved to be the most effective strategy under the conditions of this study. As an autochthonous microbial strain, STP8 represents a promising tool for sustainable agriculture by enhancing crop quality while reducing reliance on chemical inputs and may contribute to improved crop resilience to environmental stresses associated with climate change.

Practically, a single application at planting proved to be the most effective strategy for enhancing the accumulation of bioactive compounds, indicating that optimized application timing may improve the efficacy of *Trichoderma*-based biostimulants, while avoiding unnecessary repeated applications. Further research integrating physiological, biochemical, and molecular analyses is required to elucidate the mechanisms by which the native *Trichoderma* sp. strain STP8 regulates the biosynthesis of bioactive compounds in lettuce.

## Figures and Tables

**Table 1:** Experimental variants of *Trichoderma* sp. strain STP8 suspension application.

Number of Applications	Variant Mark	Application Time
STP8 application omitted	Control	untreated plants from natural/untreated seeds
STP8 applied once	A1	at seed sowing
	B1	at planting of seedlings from untreated seeds
	C1	26 DAP seedlings from untreated seeds
STP8 applied twice	A2	at seed sowing and at planting of seedlings
	B2	at planting of seedlings (from untreated seeds) and 26 DAP
	C2	at seed sowing and 26 DAP
STP8 applied three times	A3	at seed sowing, at planting of seedlings, and 26 DAP

## Data Availability

The original contributions presented in this study are included in the articlel. Further inquiries can be directed to the corresponding author(s).

## Abbreviations

The following abbreviations are used in this manuscript:

DAP	Days after planting
Chl a	Chlorophyll a
Chl b	Chlorophyll b
T chl	Total chlorophyll
T car	Total carotenoids
TPC	Total phenolic compound content
TNFC	Total non-flavonoid compound content
TFC	Total flavonoid content
ABTS	2,2′-azino-bis(3-ethylbenzothiazoline-6-sulfonic acid)
DPPH	2,2-diphenyl-1-picrylhydrazyl
FRAP	Ferric Reducing Antioxidant Power
